# Understanding Adherence to Daily and Intermittent Regimens of Oral HIV Pre-exposure Prophylaxis Among Men Who Have Sex with Men in Kenya

**DOI:** 10.1007/s10461-014-0958-x

**Published:** 2014-11-29

**Authors:** Peter Mwangi Mugo, Eduard J. Sanders, Gaudensia Mutua, Elisabeth van der Elst, Omu Anzala, Burc Barin, David R. Bangsberg, Frances H. Priddy, Jessica E. Haberer

**Affiliations:** 1 Center for Geographic Medicine - Coast, Kenya Medical Research Institute, Kilifi, Kenya; 2 Nuffield Department of Clinical Medicine, University of Oxford, Headington, UK; 3 Kenya AIDS Vaccine Initiative - Institute of Clinical Research, University of Nairobi, Nairobi, Kenya; 4 The EMMES Corporation, Rockville, MD USA; 5 Massachusetts General Hospital, Boston, MA USA; 6 Harvard Medical School, Boston, MA USA; 7 International AIDS Vaccine Initiative, New York, USA

**Keywords:** HIV, Pre-exposure prophylaxis, Intermittent PrEP adherence, Men who have sex with men

## Abstract

A qualitative assessment of Kenyan men who have sex with men taking daily and intermittent oral HIV pre-exposure prophylaxis (PrEP) found stigma, sex work, mobility, and alcohol impacted adherence. We analyzed quantitative data from the same cohort to explore different definitions of intermittent adherence. Volunteers were randomized to daily emtricitabine/tenofovir or placebo, or intermittent (prescription: Mondays/Fridays/after sex, maximum 1 dose/day) emtricitabine/tenofovir or placebo (2:1:2:1), and followed for 4 months. By electronic monitoring, median adherence for daily dosing was 80 %. Median adherence for intermittent dosing was 71 % per a “*relaxed”* definition (accounting for off-prescription dosing) and 40 % per a “*strict”* definition (limited to the prescription). Factors associated with lower adherence included travel, transactional sex, and longer follow-up; higher adherence was associated with daily dosing and an income. The definition of intermittent dosing strongly affects interpretation of adherence. These findings suggest interventions should address challenges of mobility, sex work, and long-term PrEP.

**Electronic supplementary material:**

The online version of this article (doi:10.1007/s10461-014-0958-x) contains supplementary material, which is available to authorized users.

## Introduction

About 2.3 million people are newly infected with HIV every year globally [1]. In Kenya, up to 15 % of new infections occur among men who have sex with men (MSM), and the HIV prevalence among MSM (15 %) is twice that in the general population [2]. Cohort studies in Kenya have documented a very high HIV incidence among MSM. A multi-center cohort study documented an incidence of 6.8 cases per 100 person-years of observation, which was more than double the incidence among women and heterosexual men in the same cohorts [3]. In a later analysis at one of the centers, incidence among men who have sex with men exclusively was 35.2 cases per 100 person-years compared to 5.8 per 100 person-years among men who have sex with men and women [4]. Despite the high HIV burden, engagement of MSM in research and care is hampered by stigma and discrimination, and the fact that male same sex behavior is criminalized in Kenya, as in many other African countries [5]. However, interventions for MSM are now a key priority in the Kenyan national HIV strategic plan [6].

Pre-exposure prophylaxis (PrEP), the administration of antiretroviral medications (ARVs) to at-risk HIV-negative persons to prevent HIV acquisition, has been shown to be effective. In the iPrEx trial among MSM and transgender women, a 44 % relative reduction in risk of HIV acquisition was seen among participants randomized to use daily oral emtricitabine/tenofovir versus placebo [7]. Efficacy was higher in two other trials of daily emtricitabine/tenofovir: 75 % among serodiscordant heterosexual couples in the Partners PrEP trial conducted in Kenya and Uganda [8] and 62 % among heterosexual men and women in the TDF2 trial conducted in Botswana [9]. Daily Tenofovir tablets alone achieved an efficacy of 67 % in the Partners PrEP trial [8] and 49 % in the Bangkok Tenofovir study conducted among injecting drug users in Thailand [10]. In contrast, daily PrEP did not show efficacy in the FEM-PrEP and VOICE trials conducted among high-risk women in Eastern and Southern Africa countries. However, analysis of drug levels pointed to low adherence as the likely cause of the lack of efficacy [11–13]. No significant safety concerns with daily use of emtricitabine/tenofovir tablets were reported in any of the above studies. Overall, higher efficacy was consistently seen among volunteers with higher adherence. Based on the results of the iPrEx and Partners trials, the emtricitabine/tenofovir combination tablet was approved for PrEP use in the United States, and guidelines for clinical use released by the WHO [14]. In South Africa, clinical guidelines have been developed, but regulatory approval is pending [15].

Several post-trial access studies and demonstration projects are currently underway [16, 17]. A secondary analysis of the iPrEx trial showed that the greatest reduction in HIV transmission would be achieved if PrEP is targeted toward MSM and transgender women reporting unprotected receptive anal intercourse, cocaine use, or sexually transmitted infections [18]. A PrEP demonstration project in the US reported high interest in PrEP among MSM attending a sexually transmitted disease clinic [19] and an open label extension of the iPrEx study found high PrEP uptake coupled with high protection that increased with increasing levels of adherence [20]. A recent systematic review of mathematical modeling studies has found PrEP to be most cost-effective when targeted at “key populations at the highest risk of HIV exposure” [21].

To address concerns about long-term toxicity, adherence, and cost of daily oral PrEP, efficacy of less-than-daily dosing has been assessed in animal and mathematical models. In the macaque model, various forms of intermittent oral administration achieved 100 % efficacy, including 2 h before and 24 h after a weekly virus challenge [22], as well as 1, 3, or 7 days before exposure followed by a second dose 2 h after exposure [23]. A mathematical modeling study using data from the iPrEx trial showed that two doses per week would correspond to a 76 % reduction in HIV infections, while four and seven doses a week would correspond to 96 and 99 % reduction, respectively [24]. In October 2014, the IPERGAY trial announced interim results showing efficacy of intermittent PrEP, resulting in discontination of the placebo arm.

Prior to the release of results from any of the PrEP trials, a phase I/II randomized controlled trial of intermittent oral PrEP was conducted in 2009–2010 among MSM in Kenya [25]. In this trial, the prescription for intermittent dosing was to take one tablet twice weekly on Mondays and Fridays and within 2 hours after sex, occurring on other days. The study documented an excellent overall safety profile for both daily and intermittent regimens. Using the medication event monitoring system (MEMS), lower adherence was seen in the intermittent regimen compared to the daily regimen (68 vs. 83 %), particularly with regard to post-coital doses (26 %). Qualitative assessments revealed that most trial volunteers favored intermittent dosing, but that adherence to both daily and intermittent regimens was hampered by stigmatizing effects of PrEP use, sex work, mobility, and alcohol use [26].

In the primary analysis [25], intermittent adherence was defined as MEMS openings strictly per the above-noted prescription. Openings on days other than Mondays, Fridays, and on days with no reported sex were considered as non-adherence. Volunteers who would not strictly follow this relatively complex, calendar plus activity-based, regimen would have low calculated adherence. However, considering emerging evidence that efficacy of PrEP may be more dependent on overall number of doses per week [24], such individuals may still have taken PrEP in a way that affords protection against HIV. A more relaxed definition may not only predict efficacy more accurately, but it also allows for variation in individual behaviors, preferences, and challenging life circumstances. Better understanding of dosing patterns and the factors that may influence both daily and intermittent adherence will be critical in optimizing use of PrEP in real-life settings.

In the analysis presented here, we use data from the same Kenyan trial to further characterize adherence behavior by assessing factors associated with adherence to daily and intermittent regimens, and exploring the impact of different definitions of intermittent adherence.

## Methods

### Study Setting and Ethics Statement

Data for this study came from a randomized, placebo controlled blinded trial of oral PrEP (Clinical trials.gov number NCT00971230), conducted at two centers in Kenya: Kenya AIDS Vaccine Initiative [KAVI]-Kangemi in Nairobi, and KEMRI Center for Geographic Medicine Research-Coast [CGMRC] in Kilifi. The study was sponsored by the International AIDS Vaccine Initiative (IAVI) and approved by the Kenyatta National Hospital Ethics Review Committee, the Kenya Medical Research Institute National Ethics Review Committee and the Kenya Pharmacy and Poison’s Board (Ministry of Health).

### Volunteers

Trial volunteers were recruited from ongoing HIV prevention cohorts at the two centers, which enroll MSM and female sex workers [3]. Cohort volunteers attend monthly or quarterly clinic visits where they access HIV testing and risk reduction counseling, male condoms and water-based lubricants, screening and treatment of sexually transmitted infections, and referral for adult male circumcision for men practicing heterosexual sex. Volunteers with at least 3 months of follow-up in the cohorts were enrolled. In Kilifi, five women were included to avoid community identification of the study as MSM-exclusive. However, women were excluded from the current analysis given the significant socio-behavioral differences compared to MSM.

### Study Procedures

Detailed procedures for the trial are described elsewhere [25]. Briefly, volunteers were randomized to either daily self-administration of oral emtricitabine/tenofovir or placebo, or intermittent self-administration of oral emtricitabine/tenofovir or placebo (in a 2:1:2:1 ratio), and followed monthly for 4 months. Adherence data were collected by the Medication Event Monitoring System (MEMS), in which each opening of the pill bottle was recorded electronically on the bottle cap, and by a face-to-face timeline followback interview calendar (FBIC), in which pill taking in the prior month was reviewed during an interview [27]. Volunteers were given a key chain pill holder in which they were instructed to load a few pills when they were unable or did not wish to carry the MEMS bottle. Curiosity openings (i.e., bottle openings with no pills taken out) and pocket doses (i.e., extra pills taken out for carrying in the pill holder) were assessed at the FBIC interviews. To assess adherence to post-coital doses, sexual activity data were collected via daily Short Message Service (SMS) queries, as well as through the FBIC.

### Definitions and Statistical Analyses of Adherence

Monthly adherence to daily regimen was calculated as the number of MEMS openings (i.e., number of doses taken) divided by 28 (i.e., the number of doses expected over the monitoring period). Monthly adherence to intermittent regimen was calculated by two methods: *strict* i.e. per prescription, and *relaxed* i.e. allowing some off-prescription doses (Table 1). Off-prescription dosing could occur, for example, if a volunteer took a forgotten Monday dose on a Tuesday on which sex did not occur. The relaxed adherence calculation counts that dose in the numerator, while the strict adherence ignores it. Hence when more doses are taken off-prescription, the relaxed method results in higher adherence compared to the strict method. For the primary analyses of this paper the relaxed definition of intermittent adherence was used, while for secondary analyses the strict definition was used.

**Table 1:** Formulas used in calculation of different monthly adherence measures based on 28-day monitoring periods

$${\text{Adherence to daily dosing}}\; = \; \frac{\text{Total number of MEMS openings}}{{28\;{\text{days}}}}$$
$${\text{Strict adherence to intermittent dosing}}\; = \;\frac{{\begin{array}{*{20}c} {\left( {\text{Number of MEMS openings on Mondays and Fridays}} \right) \; + \;({\text{Number of}}} \\ {{\text{MEMS openings on other days on which sex was reported by FBIC}}^{\text{a}} )} \\ \end{array} \; }}{{\begin{array}{*{20}c} {({\text{Mondays and Fridays}}) + ({\text{Non - Mondays and Non - Fridays on which}}} \\ {{\text{sex was reported by FBIC}}^{\text{a}} )} \\ \end{array} }}$$
$${\text{Relaxed adherence to intermittent dosing}} = \frac{\text{Total number of MEMS openings}}{{\begin{array}{*{20}c} {\left( {\text{Mondays and Fridays}} \right) + ({\text{Non - Mondays and Non - Fridays}}\,{\text{on which}}} \\ {{\text{sex was reported by FBIC}}^{\text{a}} )} \\ \end{array} }}$$

^a^Follow back interview calendar (FBIC) sexual activity data were used in preference to SMS data due to concerns about the reliability of SMS data resulting from frequent mobile network outages during the study period

Although the denominators are different for the intermittent and daily adherence measures, the use of percent adherence allowed for comparative and combined analyses. We compared overall adherence levels over the 4-month period among treatment and dosing groups using the Wilcoxon rank-sum test. Using descriptive methods, we calculated the number of volunteers with >80 % overall adherence, a cut-off that has been used in prior studies to categorize adherence, i.e. low or high, though not a firm efficacy threshold [28]. Counting total number of MEMS openings, corresponding to the relaxed definition for intermittent dosing, we calculated the proportion of volunteers who took four or more doses per week and the median number of doses taken per week.

Potential factors associated with monthly adherence over the four-month study period were examined via univariable and multivariable linear repeated measures models. Data from all volunteers regardless of regimen were assessed together. Baseline variables evaluated were: dosing regimen (daily versus intermittent), age, years of education, source of income, financial care of dependents, engagement in sex work, and sex with men in month prior to enrollment. Variables evaluated as time-varying covariates (measured monthly over the four-month follow-up period) were: any sex, number of sexual partners, any occurrence of sex while drunk, sex with a new partner, <100 % condom use with new or HIV + partners, involvement in transactional sex, receptive or insertive anal intercourse or <100 % condom use with anal intercourse, frequent travel (more than three nights on average per week), any alcohol use, any drug use, and duration of follow-up.

All variables with *p* < 0.10 from the univariable model were included in an initial multivariable model. Subsequently, variables with *p* ≥ 0.10, except dosing regimen in which we had an a priori interest, were excluded, the model was then re-fit and all interactions examined. A two-sided *p* value of <0.05 was considered to indicate statistical significance. Statistical analyses were performed with the use of SAS software, version 9.2 (SAS Institute, Cary, NC, USA).

## Results

Sixty-two MSM (34 in Nairobi and 28 in Kilifi) who had at least 1 month of follow-up in the trial were included in the analyses. Table 2 presents their characteristics at enrolment and during the four-month follow-up period.

**Table 2:** Enrolment and follow-up characteristics of volunteers randomized to daily versus intermittent dosing

Characteristics at enrolment	Daily	Intermittent
Number of volunteers	29	33
Mean age in years (range)	26 (20–38)	26 (18–35)
Mean years of education (range)	10 (0–16)	10 (0–15)
Source of income		
Self (%)	62	61
Family (%)	24	15
No employment (%)	14	24
Married, monogamous (%)	0	6
Engaged in sex work in past month (%)	48	48
Engaged in sex with men in past month (%)	83	88

Characteristics during follow-up^a^		
Any sex (%)	89	94
Any sex while drunk (%)	36	44
Sex with a new partner (%)	65	65
<100 % condom use with new or HIV + partners (%)	34	17
Involved in transactional sex (%)	63	63
Sex with a known HIV + person (%)	1	2
Receptive anal intercourse (%)	54	60
Insertive anal intercourse (%)	62	60
<100 % condom use with anal intercourse (%)	36	34
Frequent travel (%)	40	44
Any alcohol use (%)	54	69
Any drug use (%)	40	52

^a^Average event rate per month, based on events reported for 114 and 126 follow-up months for daily and intermittent groups, respectively

### Adherence by Treatment and Dosing Group

Because there was no difference in levels of adherence between placebo and active treatment groups (data not shown), the two were combined for all subsequent analyses.

Median [interquartile range, IQR] adherence over the four-month follow-up period was 80 % [63–88 %] for the daily dosing group and 71 % [51–99 %] for the intermittent dosing group, using the relaxed definition (*p* = 0.81). Using the strict definition, adherence to intermittent dosing was 40 % [21–51 %] (*p* < 0.0001, comparing with the daily dosing group).

Fifteen (52 %) volunteers in the daily group had >80 % adherence. In the intermittent group, 14 (42 %) volunteers had adherence >80 % under relaxed definition, and under strict definition only one volunteer (3 %) had adherence >80 %. The proportion of volunteers with four or more doses per week was 86 % (25/29) in the daily group and 3 % (1/33) in the intermittent group. Median MEMS openings per week was 5.6 [4.4–6.1] in the daily group and 2.1 [1.3–2.7] in the intermittent group.

### Factors Associated with PrEP Adherence

In the multivariate model combining daily and relaxed intermittent adherence, lower adherence was significantly associated with frequent travel in the past month and marginally associated with transactional sex in the past month (Table 3). Dosing regimen (daily versus intermittent) did not show any significant association with PrEP adherence.

**Table 3:** Factors associated with adherence to PrEP for combined daily and relaxed intermittent dosing groups

Factor	Univariable analysis		Multivariable analysis	
	Estimate	*p* value	Estimate	*p* value
Age (per year)	1.0	0.13	–	–
Years of education	0.6	0.46	–	–
Source of income		0.48	–	–
Self	9.6	0.23	–	–
Family	6.0	0.54	–	–
No employment	Reference	N/A	–	–
Financial care of dependents	3.0	0.68	–	–
Engaged in sex work in month prior to enrollment	−7.2	0.24	–	–
Had sex with men in month prior to enrollment	0.9	0.92	–	–
Any sex^a^	−19.4	0.02	–	–
# Sexual partners^a^	0.2	0.72	–	–
Any occurrence of sex while drunk^a^	−6.2	0.18	–	–
Sex with a new partner^a^	1.6	0.71	–	–
<100 % condom use with new or HIV + partners^a^	1.2	0.80	–	–
Involved in transactional sex^a^	−8.7	0.08	−8.7	0.07
Receptive anal intercourse^a^	−4.1	0.42	–	–
Insertive anal intercourse^a^	4.6	0.35	–	–
<100 % condom use with anal intercourse^a^	−2.7	0.60	–	–
Frequent travel^a^	−9.8	0.01	−9.2	0.01
Any alcohol use^a^	−0.8	0.86	–	–
Any drug use^a^	−4.8	0.38	–	–
Duration on study (per study month)^a^	−4.3	0.01	–	–
Daily Dosing Regimen^b^	2.2	0.72	5.9	0.30

Estimates should be interpreted as percentage point changes from the reference factor

^a^Time-varying covariate (measured monthly)

^b^Daily dosing regimen was retained in the multivariate model due to a priori interest

In the secondary analysis, where the strict definition for intermittent adherence was used (Table 4), transactional sex in the past month, and longer time on study (per each additional month of follow-up) were associated with lower adherence; a marginal association was also seen with frequent travel in the past month. Daily dosing (versus intermittent dosing) and self/family income (versus no employment) were associated with higher adherence; a marginal association was also seen with sex with a new partner in the past month.

**Table 4:** Factors associated with adherence to PrEP for combined daily and strict intermittent dosing groups

Factor	Univariable analysis		Multivariable analysis	
	Estimate	*p* value	Estimate	*p* value
Age (per year)	0.7	0.35	–	–
Years of education	0.8	0.41	–	–
Source of income		0.04		0.03
Self	20.1	0.02	19.0	0.01
Family	22.6	0.03	18.8	0.04
No employment	Reference	N/A	Reference	N/A
Financial Care of Dependents	−8.2	0.30	–	–
Engaged in sex work in month prior to enrollment	−6.3	0.35	–	–
Had sex with men in month prior to enrollment	−1.8	0.84	–	–
Any sex^a^	−8.9	0.19	–	–
# Sexual partners^a^	0.7	0.17	–	–
Any occurrence of sex while drunk^a^	−8.8	0.03	–	–
Sex with a new partner^a^	5.9	0.09	6.1	0.08
<100 % condom use with new or HIV + partners^a^	1.4	0.74	–	–
Involved in transactional sex^a^	−7.2	0.10	−10.2	0.02
Receptive anal intercourse^a^	−5.3	0.25	–	–
Insertive anal intercourse^a^	7.2	0.10	–	–
<100 % condom use with anal intercourse^a^	−2.2	0.64	–	–
Frequent travel^a^	−7.4	0.02	−5.1	0.09
Any alcohol use^a^	−3.3	0.41	–	–
Any drug use^a^	−4.4	0.41	–	–
Duration on study (per study month)^a^	−4.6	0.0003	−3.2	0.02
Daily Dosing Regimen^b^	25.9	<0.0001	26.7	<0.0001

Estimates should be interpreted as percentage point changes from the reference factor

^a^Time-varying covariate (measured monthly)

^b^Daily dosing regimen was retained in the multivariate model due to a priori interest

The large difference in adherence between dosing groups in the secondary analysis prompted further analysis of each dosing group separately to determine if factors may be uniquely associated with adherence to each dosing regimen (see Supplementary Tables I to III). In the multivariate model for the daily dosing group only, lower adherence was significantly associated with frequent travel (*p* = 0.01) and marginally associated with any occurrence of sex while drunk (*p* = 0.06). Frequent travel in the past month was the only factor marginally associated with a lower adherence to intermittent PrEP, using the relaxed definition (*p* = 0.07). Using the strict definition, lower adherence to intermittent PrEP was associated with transactional sex in the past month (*p* = 0.03) and longer time on study (*p* = 0.03), while higher adherence was associated with self/family income (versus no employment) (*p* = 0.04). A summary of factors associated with PrEP adherence is presented in Table 5.

**Table 5:** Summary of factors associated with PrEP adherence for different dosing groups and adherence definitions

	Dosing group and adherence definition				
	Combined daily and relaxed intermittent	Combined daily and strict intermittent	Daily only	Relaxed intermittent only	Strict intermittent only
Factors associated with higher adherence					
Daily dosing (vs intermittent dosing)	**–**	↑*	n/a	n/a	n/a
Self/family income (vs no employment)	**–**	↑*	**–**	**–**	↑*
New sex partner	**–**	**↑**	**–**	**–**	**–**
Factors associated with lower adherence					
Frequent travel	↓*	**↓**	↓*	**↓**	**–**
Transactional sex	**↓**	↓*	**–**	**–**	↓*
Longer duration of follow-up (per study month)	**–**	↓*	**–**	**–**	↓*
Sex while drunk	**–**	**–**	**↓**	**–**	**–**

↑* denotes significantly higher adherence (*p* < 0.05), ↑ denotes marginally higher adherence (*p* < 0.1), ↓* denotes significantly lower adherence, ↓ denotes marginally lower adherence, “–” denotes no association, and “*n*/*a*” means not applicable

## Discussion

In this analysis of a randomized clinical trial of daily and intermittent PrEP among Kenyan MSM, we found that the definition of intermittent adherence significantly affects interpretation of adherence behavior. As in our previous report of this trial, intermittent adherence as defined strictly by the prescribed regimen was significantly lower than daily adherence. No significant difference, however, was seen when a relaxed definition of intermittent dosing was used. Other factors associated with PrEP adherence varied to some extent depending on the definition of intermittent adherence; however, frequent travel and transactional sex consistently appeared as factors associated with lower adherence. Using the strict definition, longer duration of PrEP use was also associated with lower adherence, and a source of income (versus no employment) was associated with higher adherence.

Similar to our earlier qualitative assessments [26], the current analysis suggests that adherence interventions should address challenges related to mobility, sex work, and long-term PrEP. The best design for interventions to tackle these challenges is not clear. One potential approach would be to use counseling tailored to mobility, sex work, and long-term PrEP. Tailored counseling has been used in clinical trials [29, 30] and appears promising, but strategies for “real world” settings need further development. Effects of frequent travel could also be addressed by distributing PrEP widely so that MSM can access emergency supply when away from their usual residence.

This analysis highlights the complexities in defining and measuring adherence to an intermittent PrEP regimen. A greater number of significant factors were seen with the strict definition, which may reflect the effects of individual behaviors, preferences and circumstances that make adherence more difficult. The relaxed adherence measure likely accounts for strategies used by volunteers to take PrEP despite the challenges they face. For example, they may have opened the MEMS bottle on non-prescribed days while loading pills into keychain holders. Off-prescription dosing may also have occurred due to forgetting or temporary lack of access to the medication, leading to late dosing on a non-prescribed day. Moreover, the relaxed definition also accounts for possible limitations of the self-report follow-back interview in ascertaining the exact days on which sex occurred (i.e., some of the “off prescription” doses may actually have been taken on days when sex occurred, but day of sex was not reported accurately). An increase in sex reporting over the study period was noted in our previous paper and may explain the decrease in strict intermittent adherence seen with longer duration on study [25].

A mathematical modelling study based on data from the iPrEx trial showed that protection from HIV infection may be correlated with a specific number of PrEP doses per week, regardless of exact dosing intervals [24]. Extrapolating this model to our trial data, 86 % of volunteers in the daily group and 3 % in the intermittent group would have received 99 % protection. This extrapolated finding is concerning with regard to the intermittent dosing group. It is also important to note that achieving four doses a week, given the prescription based on sexual activity, would require sex 2 days per week on a non-Monday and non-Friday. Clinical efficacy data from the ongoing IPERGAY and ADAPT [31, 32] studies will be essential in determining the efficacy of various intermittent PrEP regimens at various adherence levels.

This study has limitations. First, our sample may have been too small to identify all the factors that may influence PrEP adherence. The factors we identified in this analysis were largely the same as those identified through qualitative assessment [26], although the comparison is limited by use of a strict definition of adherence in selecting the participants for that study. Second, the trial did not collect data on some of the factors that may have an impact on PrEP adherence, such as stigma, depression and risk perception; hence, these were not included in the regression analysis. Third, our findings with regard to the level of adherence observed may not be generalizable to settings where PrEP will be used for a longer time or where the efficacy of PrEP is known. For example, transactional sex may be associated with better post-coital adherence if PrEP users are aware that it would protect them. Lastly, our understanding of intermittent adherence using a relaxed definition was based on hypothesized use patterns in volunteers instructed to follow a strict regimen; hence, this measure may not predict the actual adherence levels if the initial prescription was relaxed as well (i.e. if PrEP users were told to take it when they could, as close as possible to a twice weekly and post-coital regimen).

In conclusion, we found that while the definition of intermittent dosing has a strong effect on interpretation of adherence behavior, results confirm earlier qualitative findings and suggest that adherence interventions should address challenges related to sex work, mobility, and long-term PrEP. As researchers consider less frequent dosing to improve adherence, careful assessment of adherence behavior is needed.

## Electronic supplementary material

Below is the link to the electronic supplementary material.

Supplementary material 1 (DOC 48 kb)
