# Supplementary material for: Understanding Adherence to Daily and Intermittent Regimens of Oral HIV Pre-exposure Prophylaxis Among Men Who Have Sex with Men in Kenya
Source: AIDS Behav. 2014 Nov 29;19(5):794–801. doi: 10.1007/s10461-014-0958-x (PMC4415948; doi:10.1007/s10461-014-0958-x)
Supplement: Supplementary file 1 — Supplementary material 1 (DOC 48 kb) [file 10461_2014_958_MOESM1_ESM.doc]

**Supplementary Table I. Secondary analysis of factors associated with adherence to daily PrEP only.**

|  | **Univariable analysis** | | **Multivariable analysis** | |
| --- | --- | --- | --- | --- |
| **Factor** | **Estimate** | **P Value** | **Estimate** | **P Value** |
| Age (per year)  Years of education  Source of income  Self  Family  No employment  Financial Care of Dependents  Engaged in sex work in month prior to enrollment  Had sex with men in month prior to enrollment  Any sex[[1]](#footnote-2)  # Sexual partners^a^  Any occurrence of sex while drunk^a^  Sex with a new partner^a^  <100% condom use with new or HIV+ partners^a^  Involved in transactional sex^a^  Receptive anal intercourse^a^  Insertive anal intercourse^a^  <100% condom use with anal intercourse^a^  Frequent travel^a^  Any alcohol use^a^  Any drug use^a^  Duration on study (per study month) ^a^ | 0.59  - 0.46  20.54  7.86  Reference  17.81  - 6.82  - 12.79  - 8.02  0.33  - 13.54  6.49  - 1.44  - 2.29  - 6.15  3.45  - 9.46  - 13.31  - 9.28  4.06  - 3.72 | 0.44  0.68  0.09  0.06  0.51  N/A  0.13  0.36  0.18  0.32  0.53  0.01  0.20  0.78  0.70  0.27  0.55  0.12  0.001  0.04  0.48  0.01 | --  --  --  --  --  --  --  --  --  --  -9.5  --  --  --  --  --  --  - 10.9  --  --  -- | --  --  --  --  --  --  --  --  --  --  --  0.06  --  --  --  --  --  --  0.01  --  --  -- |

**Supplementary Table II. Secondary analysis of factors associated with adherence to relaxed intermittent PrEP only.**

|  | **Univariable analysis** | | **Multivariable analysis** | |
| --- | --- | --- | --- | --- |
| **Factor** | **Estimate** | **P Value** | **Estimate** | **P Value** |
| Age (per year)  Years of education  Source of income  Self  Family  No employment  Financial Care of Dependents  Engaged in sex work in month prior to enroll.  Had sex with men in month prior to enroll.  Any sex[[2]](#footnote-3)  # Sexual partners^a^  Any occurrence of sex while drunk^a^  Sex with a new partner^a^  <100% condom use with new or HIV+ partners^a^  Involved in transactional sex^a^  Receptive anal intercourse^a^  Insertive anal intercourse^a^  <100% condom use with anal intercourse^a^  Frequent travel^a^  Any alcohol use^a^  Any drug use^a^  Duration on study (per study month) ^a^ | 1.15  1.65  2.53  10.79  Reference  -0.59  - 9.27  18.30  -36.51  - 0.77  1.22  - 1.48  1.97  - 12.67  1.05  6.83  - 0.55  - 10.37  11.87  - 11.79  - 4.86 | 0.26  0.19  0.78  0.83  0.49  N/A  0.95  0.33  0.21  0.01  0.50  0.87  0.82  0.81  0.09  0.90  0.35  0.94  0.07  0.14  0.21  0.10 | --    --  --  --  --  --  --  --  --  --  --  --  --  --  --  --  - 10.37  --  --  -- | --  --  --  --  --  --  --  --  --  --  --  --  --  --  --  --  --  --  0.07  --  --  -- |

**Supplementary Table III. Secondary analysis of factors associated with adherence to strict intermittent PrEP only.**

|  | **Univariable analysis** | | **Multivariable analysis** | |
| --- | --- | --- | --- | --- |
| **Factor** | **Estimate** | **P Value** | **Estimate** | **P Value** |
| Age (per year)  Years of education  Source of income  Self  Family  No employment  Financial Care of Dependents  Engaged in sex work in month prior to enroll.  Had sex with men in month prior to enroll.  Any sex[[3]](#footnote-4)  # Sexual partners^a^  Any occurrence of sex while drunk^a^  Sex with a new partner^a^  <100% condom use with new or HIV+ partners^a^  Involved in transactional sex^a^  Receptive anal intercourse^a^  Insertive anal intercourse^a^  <100% condom use with anal intercourse^a^  Frequent travel^a^  Any alcohol use^a^  Any drug use^a^  Duration on study (per study month) ^a^ | 0.27  1.87  17.7  34.5  Reference  - 6.64  - 7.22  16.47  - 4.74  - 0.26  - 0.72  4.88  -0.71  - 15.02  - 0.12  6.91  0.93  - 3.43  8.54  - 10.34  - 5.38 | 0.77  0.10  0.03  0.07  0.01  N/A  0.46  0.40  0.21  0.62  0.76  0.90  0.26  0.90  0.01  0.99  0.22  0.88  0.40  0.15  0.20  0.01 | --    20.0  31.5  Reference  --  --  --  --  --  --  --  --  -12.3  --  --  --  --  --  --  -4.3 | --  --  0.04  0.04  0.02  N/A  --  --  --  --  --  --  --  --  0.03  --  --  --  --  --  --  0.03 |

1. Time-varying covariate (measured monthly) [↑](#footnote-ref-2)
2. Time-varying covariate (measured monthly) [↑](#footnote-ref-3)
3. Time-varying covariate (measured monthly) [↑](#footnote-ref-4)
